# Corrigendum: ChEC-seq kinetics discriminates transcription factor binding sites by DNA sequence and shape *in vivo*

**DOI:** 10.1038/ncomms10264

**Published:** 2015-12-16

**Authors:** Gabriel E. Zentner, Sivakanthan Kasinathan, Beibei Xin, Remo Rohs, Steven Henikoff

*Nature Communications* 6, Article number: 8733 10.1038/ncomms9733 (2015); Published 10222015; Updated 12162015

An incorrect version of the Supplementary Data 1, in which data in columns E, F, G, H and K of the sheet ‘Reb1' were sorted independently from the other columns, was inadvertently published with this Article. This version was not used in any of the analyses presented in the paper. The HTML has now been updated to include the correct version of Supplementary Data 1.

