# Supplementary material for: ChEC-seq kinetics discriminates transcription factor binding sites by DNA sequence and shape in vivo
Source: Nat Commun. 2015 Oct 22;6:8733. doi: 10.1038/ncomms9733 (PMC4618392; doi:10.1038/ncomms9733)
Supplement: Supplementary Information — Supplementary Figures 1-9, Supplementary Tables 1-2 and Supplementary References [file ncomms9733-s1.pdf]

Supplementary Figures

Supplementary Figure 1

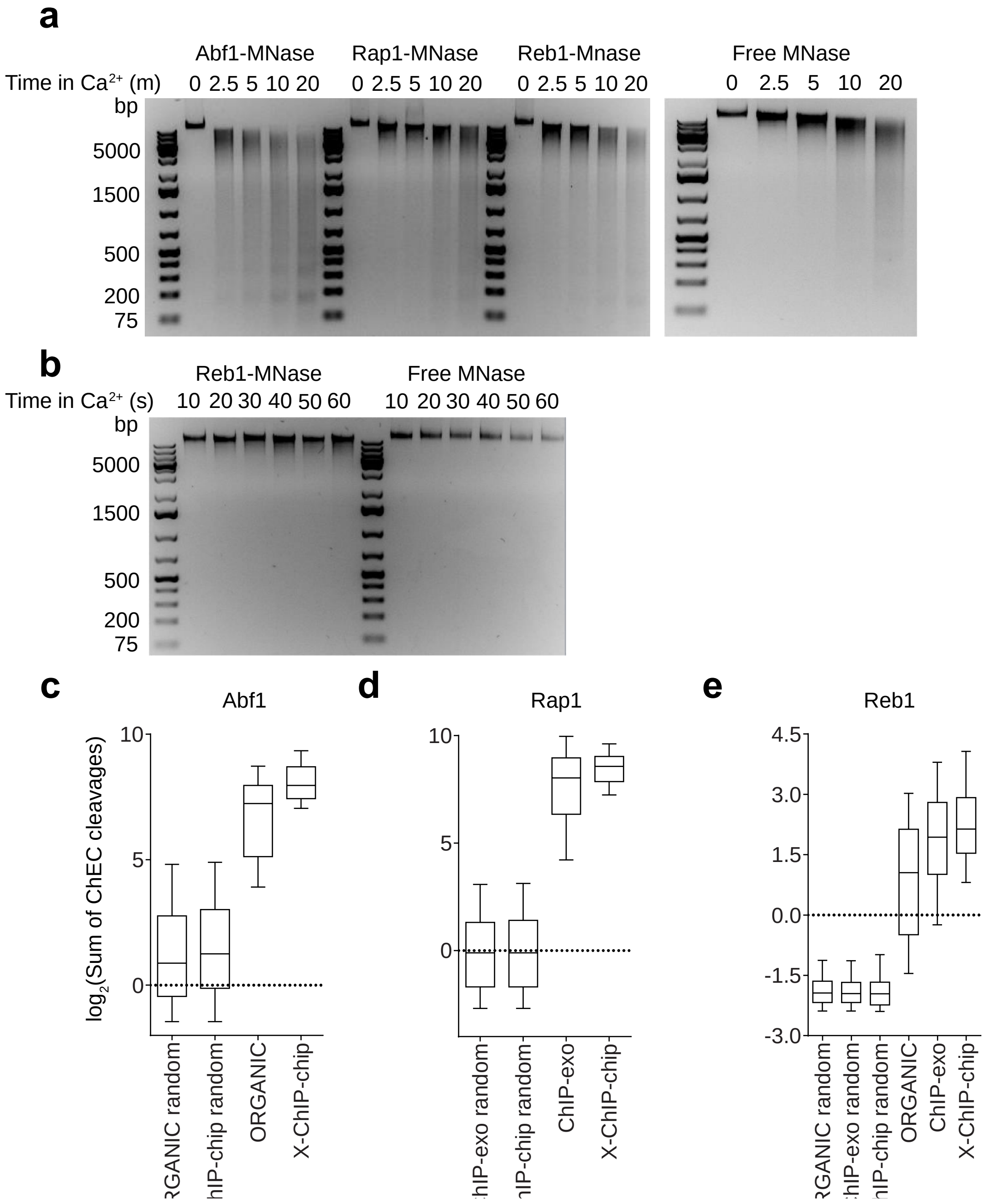

Supplementary Figure 1. Specificity of ChEC-seq

(**a**) Agarose gel analysis of genomic DNA from TF-MNase and free MNase strains 0, 2.5, 5, 10, and 20 m after calcium addition. (**b**) Agarose gel analysis of genomic DNA from Reb1-MNase and free MNase strains 10, 20, 30, 40, 50, and 60 s after calcium addition. Also shown are boxplots of total ChEC-seq cleavages at peaks previously determined by various ChIP methods and an equal number of random sites for (**c**) Abf1, (**d**) Rap1, and (**e**) Reb1.

Supplementary Figure 2

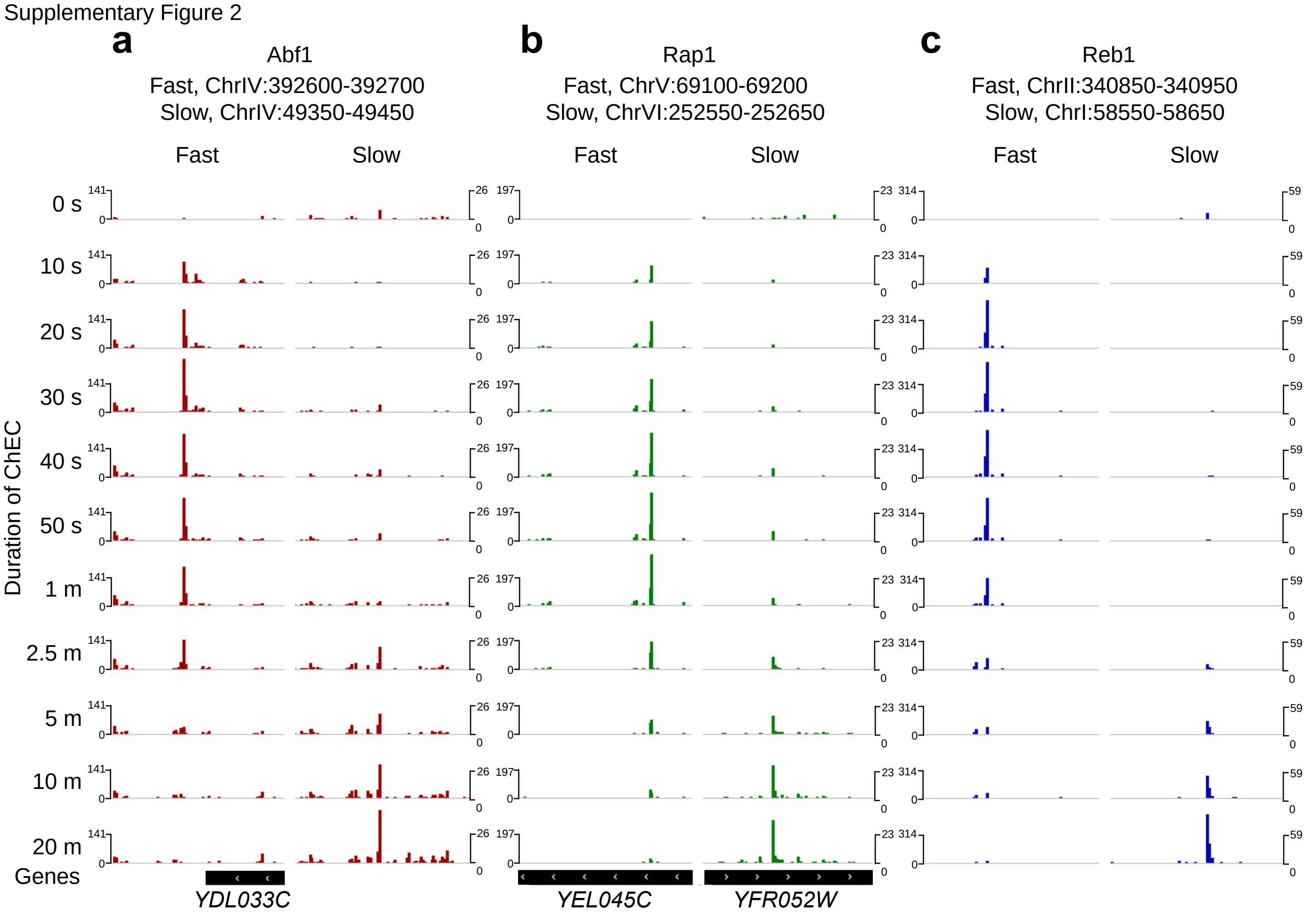

Supplementary Figure 2. Fast and slow ChEC-seq sites

Tracks of ChEC-seq signal at a fast and slow site for (**a**) Abf1, (**b**) Rap1, and (**c**) Reb1.

Supplementary Figure 3

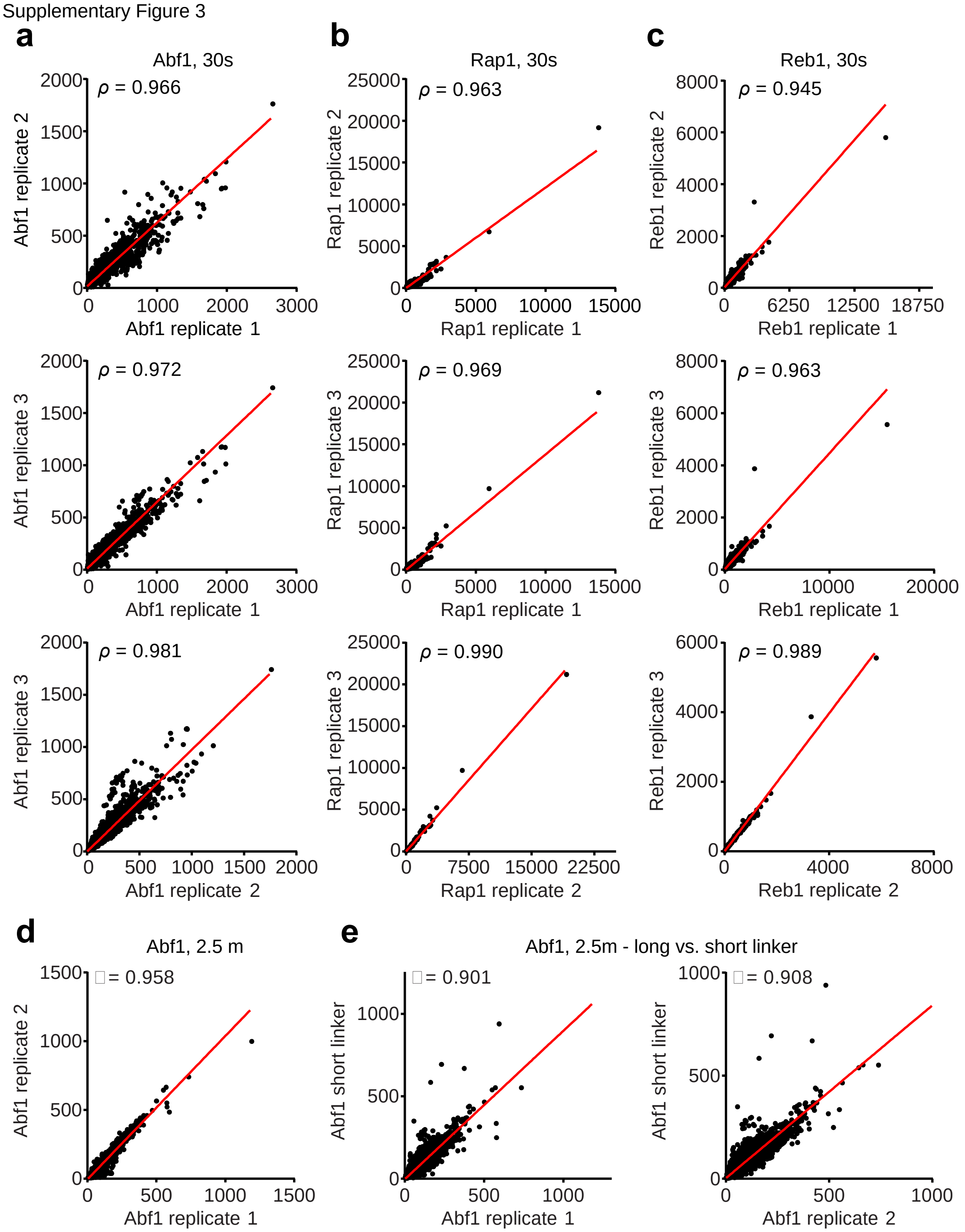

Supplementary Figure 3. ChEC-seq peaks are highly reproducible

Pairwise correlations of 30 s ChEC-seq replicate signal at (**a**) Abf1, (**b**) Rap1, and (**c**) Reb1 peaks. (**d**) Pairwise correlation of 2.5 m ChEC-seq replicate signal. (**e**) Pairwise correlations of 2.5 m Abf1 ChEC-seq replicate signal with 2.5 m Abf1 short linker ChEC-seq signal. The sum of cleavages in a 50 bp window around each peak midpoint was taken to be that peak's occupancy. The Spearman's rank correlation coefficient ρ for each pairwise comparison is reported.

Supplementary Figure 4

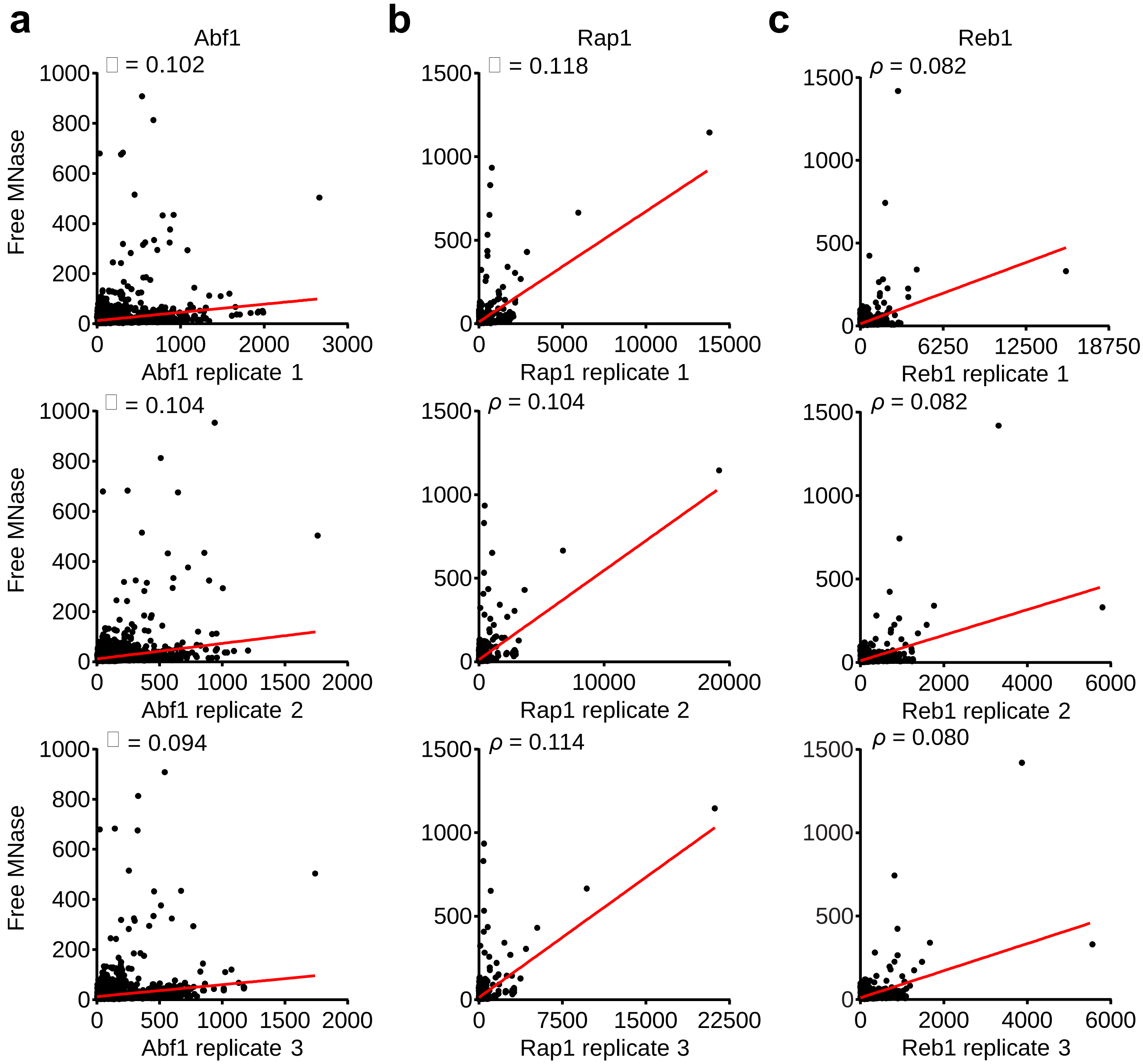

Supplementary Figure 4. ChEC-seq peaks show poor correlation with free MNase signal

Pairwise correlations of 30 s ChEC-seq and free MNase signal at (**a**) Abf1, (**b**) Rap1, and (**c**) Reb1 peaks. The sum of cleavages in a 50 bp window around each peak midpoint was taken to be that peak's occupancy. The Spearman's rank correlation coefficient ρ for each pairwise comparison is reported.

Supplementary Figure 5

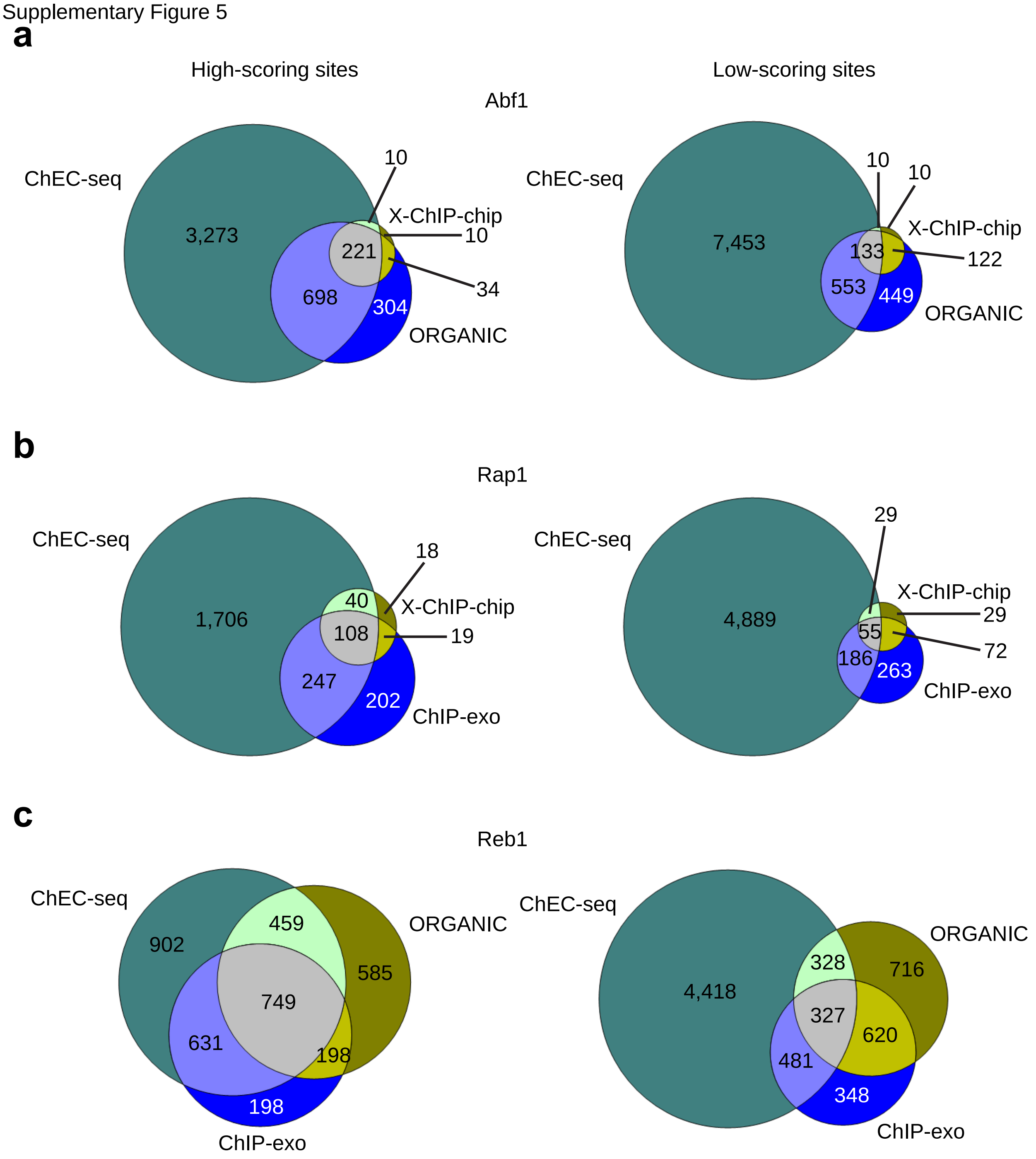

Supplementary Figure 5. Overlap of ChEC-seq peaks with ChIP peaks

(**a**) Venn diagrams of overlap between high- and low-scoring Abf1 ChEC-seq sites with Abf1 X-ChIP-chip and ORGANIC peaks. (**b**) Venn diagrams of overlap between high- and low-scoring Rap1 ChEC-seq sites with Rap1 X-ChIP-chip and ChIP-exo peaks. (**c**) Venn diagrams of overlap between high- and low-scoring Reb1 ChEC-seq sites with Reb1 ORGANIC and ChIP-exo peaks.

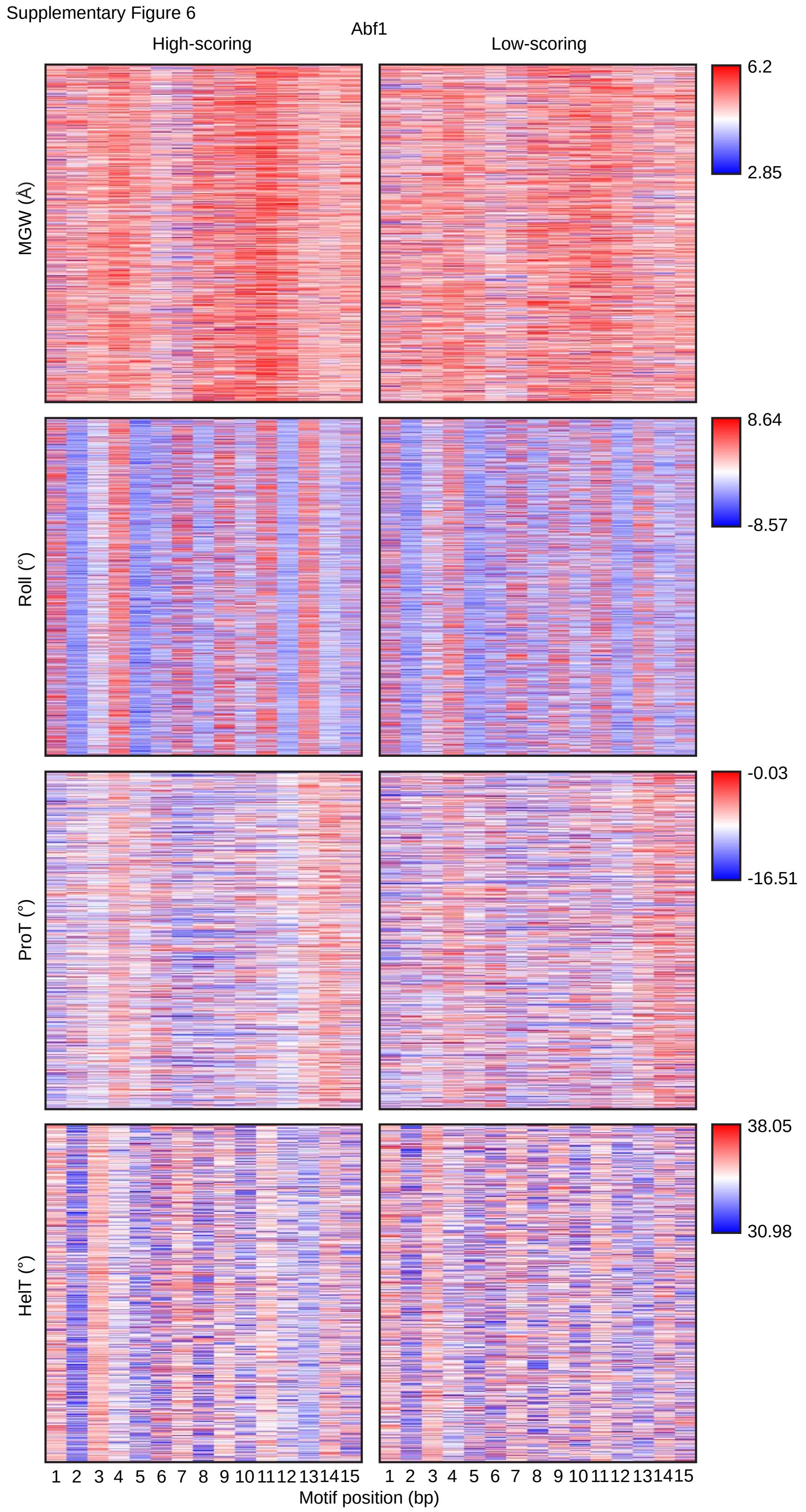

Supplementary Figure 6. High-scoring and low-scoring Abf1 sites display similar shape profiles. Heatmaps of DNA shape features minor groove width (MGW), Roll, propeller twist (ProT), and helix twist (HelT) around high- and low-scoring Abf1 sites ranked ascending by motif match *p*-values.

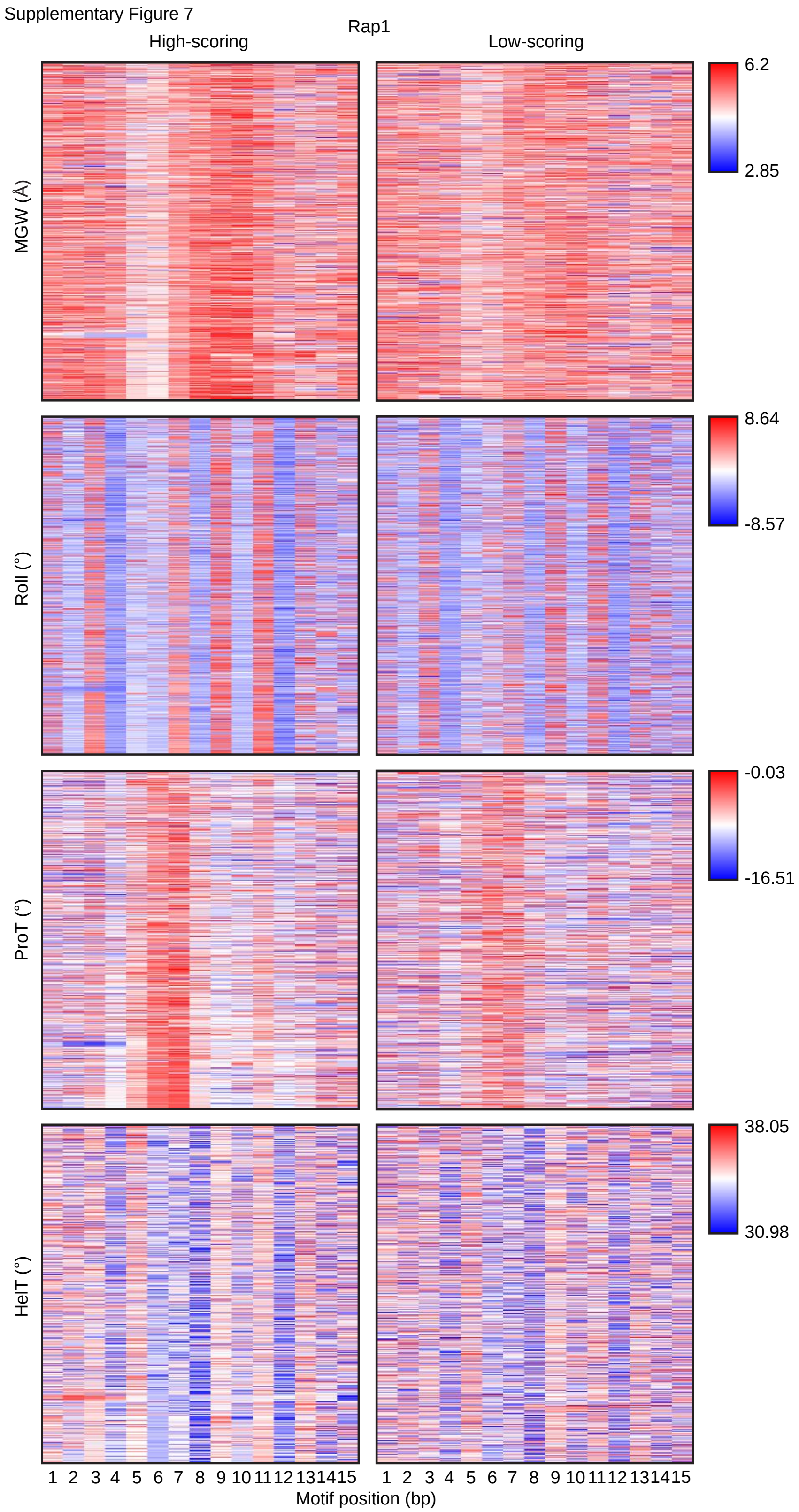

Supplementary Figure 7. High-scoring and low-scoring Rap1 sites display similar shape profiles. Heat maps of DNA shape features minor groove width (MGW), Roll, propeller twist (ProT), and helix twist (HelT) around high- and low-scoring Rap1 sites ranked ascending by motif match *p*-values.

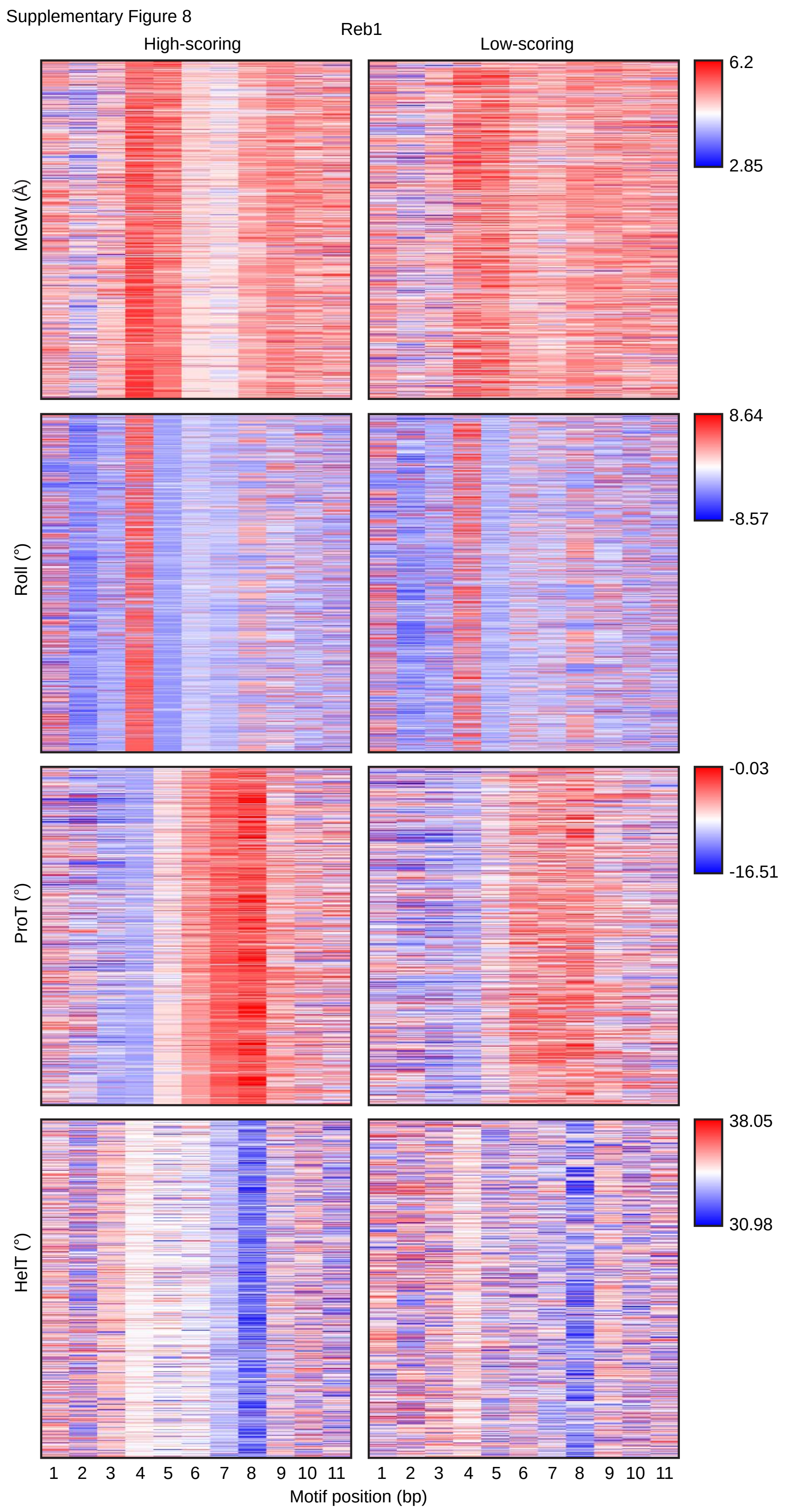

Supplementary Figure 8. High-scoring and low-scoring Reb1 sites display similar shape profiles. Heat maps of DNA shape features minor groove width (MGW), Roll, propeller twist (ProT), and helix twist (HelT) around high- and low-scoring Reb1 sites ranked ascending by motif match *p*-values.

Supplementary Figure 9

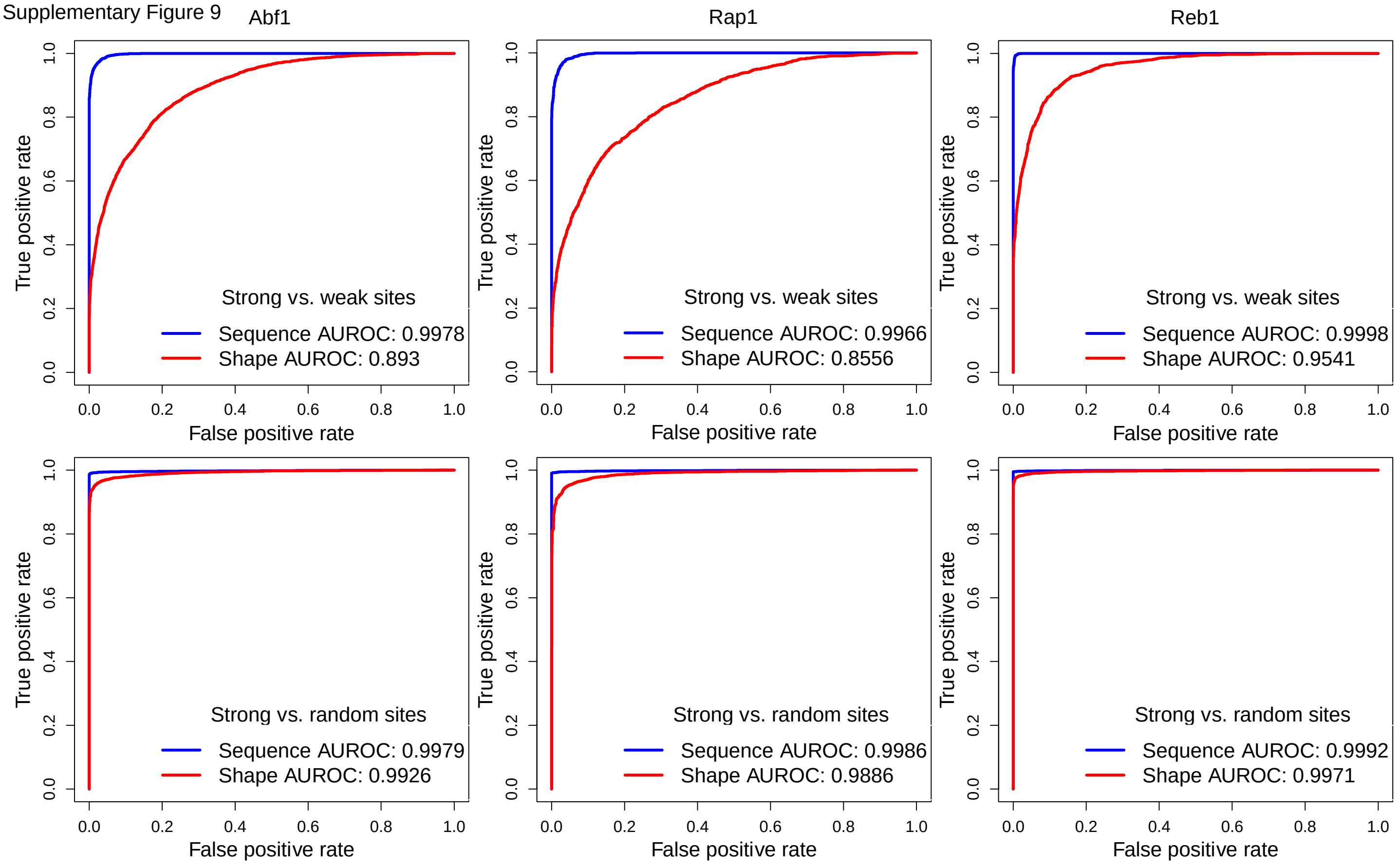

Supplementary Figure 9. Classification of high-scoring and low-scoring motif sites using DNA sequence or shape. Models based on L2-regularized multiple linear regression (MLR) encoding either sequence (blue) or shape (red) were used to distinguish sequences containing high-scoring and low-scoring motifs (top row) for Abf1 (left), Rap1 (center), and Reb1 (right). The lower AUROC values for the shape-based classification indicate the higher similarity of high-scoring and low-scoring sites in terms of DNA shape compared to sequence. This difference between sequence- and shape-based models cannot be observed when the MLR classification is applied to sequences containing high-scoring motifs and random sequences (bottom row).

Supplementary Tables

Plasmid	Description	Source
pFA6a-3HA-KanMX6	C-terminal 3xHA-tagging vector (F2/R1 compatible), kanMX6	Addgene
pFA6a-3HA-HIS3MX6	C-terminal 3xHA-tagging vector (F2/R1 compatible), HIS3MX6	Addgene
pFA6a-3HA-TRP1	C-terminal 3xHA-tagging vector (F2/R1 compatible), TRP1	Addgene
pFA6a-kanMX6	Deletion vector (F2/R1 compatible), kanMX6	Sue Biggins
pGZ108	C-terminal 3xFLAG-MNase-tagging vector (F2/R1 compatible), kanMX6	This study
pGZ109	C-terminal 3xFLAG-MNase-tagging vector (F2/R1 compatible), HIS3MX6	This study
pGZ110	C-terminal 3xFLAG-MNase-tagging vector (F2/R1 compatible), TRP1	This study
pGZ136	3xFLAG-MNase-SV40 NLS under the control of the *REB1* promoter in pRS406	This study
pGZ172	MNase-3FLAG-REB1 under the control of the *REB1* promoter in pRS413	This study
pGZ173	C-terminal short linker-MNase-tagging vector (F2/R1 compatible), kanMX6	This study
pRS406	Integrating vector, URA3	Toshio Tsukiyama
pRS413	Shuttle vector, HIS3	Toshio Tsukiyama

Supplementary Table 1. Plasmids used in this study

Strain	Genotype	Source
GZY85	*MATa ade2-1 can1-100 his3-11, 15 leu2-3, 112 trp1-1 ura3-1 RAD5+ REB1-3FLAG-MNase-kanMX6*	This study
GZY98	*MATa ade2-1 can1-100 his3-11, 15 leu2-3, 112 trp1-1 ura3-1 RAD5+ ura3::*P_{REB1}*-3FLAG-MNase-URA3*	This study
GZY99	*MATa ade2-1 can1-100 his3-11, 15 leu2-3, 112 trp1-1 ura3-1 RAD5+ ABF1-3FLAG-MNase-kanMX6*	This study
GZY100	*MATa ade2-1 can1-100 his3-11, 15 leu2-3, 112 trp1-1 ura3-1 RAD5+ RAP1-3FLAG-MNase-kanMX6*	This study
GZY112	*MATa ade2-1 can1-100 his3-11, 15 leu2-3, 112 trp1-1 ura3-1 RAD5+ ABF1-SL-MNase-kanMX6*	This study
GZY113	*MATa ade2-1 can1-100 his3-11, 15 leu2-3, 112 trp1-1 ura3-1 RAD5+ RAP1-SL-MNase-kanMX6*	This study
GZY114	*MATa ade2-1 can1-100 his3-11, 15 leu2-3, 112 trp1-1 ura3-1 RAD5+ REB1-SL-MNase-kanMX6*	This study
GZY115	*MATa ade2-1 can1-100 his3-11, 15 leu2-3, 112 trp1-1 ura3-1 RAD5+ reb1Δ::kanMX6 pGZ172 (*P_{REB1}*-MNase-3FLAG-REB1 HIS3 CEN ARS)*	This study
SKY1	*MATa ade2-1 can1-100 his3-11, 15 leu2-3, 112 trp1-1 ura3-1 RAD5+ ABF1-3FLAG-kanMX4*	Kasinathan *et al*.[1]

Supplementary Table 2. Yeast strains used in this study
All strains were constructed in the W1588-4C background, which is isogenic to W303-1A except that a weak *rad5* mutation is repaired[2].

Supplementary References

1. Kasinathan, S., Orsi, G.A., Zentner, G.E., Ahmad, K. & Henikoff, S. High-resolution mapping of transcription factor binding sites on native chromatin. *Nat Meth* **11**, 203-209 (2014).
2. Zhao, X., Muller, E.G.D. & Rothstein, R. A Suppressor of Two Essential Checkpoint Genes Identifies a Novel Protein that Negatively Affects dNTP Pools. *Mol Cell* **2**, 329-340 (1998).
